# Commercial price variation in precision oncology testing across US hospitals

**DOI:** 10.1093/haschl/qxag203

**Published:** 2026-08-06

**Authors:** Maryam Mooghali, Joshua J Skydel, Cary P Gross, Stacie B Dusetzina, Zenta Walther, Maryam Lustberg, Joseph S Ross, Joshua D Wallach

**Affiliations:** Department of Internal Medicine, Yale School of Medicine, New Haven, CT 06510, United States; Yale Collaboration for Regulatory Rigor, Integrity, and Transparency (CRRIT), Yale School of Medicine, New Haven, CT 06510, United States; Yale Collaboration for Regulatory Rigor, Integrity, and Transparency (CRRIT), Yale School of Medicine, New Haven, CT 06510, United States; Section of Rheumatology, Allergy & Immunology, Yale School of Medicine, New Haven, CT 06510, United States; Department of Internal Medicine, Yale School of Medicine, New Haven, CT 06510, United States; Cancer Outcomes Public Policy and Effectiveness Research (COPPER) Center, Yale Cancer Center, New Haven, CT 06510, United States; Department of Health Policy, Vanderbilt University School of Medicine, Nashville, TN 37203, United States; Department of Medicine, Vanderbilt-Ingram Cancer Center, Nashville, TN 37232, United States; Department of Pathology, Yale University School of Medicine, New Haven, CT 06510, United States; Department of Medicine, Yale Cancer Center, New Haven, CT 06510, United States; Department of Internal Medicine, Yale School of Medicine, New Haven, CT 06510, United States; Yale Collaboration for Regulatory Rigor, Integrity, and Transparency (CRRIT), Yale School of Medicine, New Haven, CT 06510, United States; Department of Health Policy and Management, Yale School of Public Health, New Haven, CT 06510, United States; Center for Outcomes Research and Evaluation, Yale-New Haven Health System, New Haven, CT 06510, United States; Yale Collaboration for Regulatory Rigor, Integrity, and Transparency (CRRIT), Yale School of Medicine, New Haven, CT 06510, United States; Department of Epidemiology, Rollins School of Public Health, Emory University, Atlanta, GA 30322, United States

**Keywords:** precision oncology, biomarker testing, price transparency, negotiated rates, price variation

## Abstract

**Introduction:**

Precision oncology biomarker testing informs prognosis and guides treatment selection. These tests represent a growing component of cancer care costs.

**Methods:**

We conducted a cross-sectional study of US commercial payer-negotiated rates for biomarker tests for breast, colon, and non-small cell lung cancer, using Turquoise Health hospital pricing database. Outcomes included geographically adjusted payer-negotiated rates, within- and between-hospital price variation, and differences by payer, rurality, and hospital size, assessed using generalized linear models adjusted for multiple comparisons.

**Results:**

We identified 422 515 negotiated rates for 28 biomarker tests across 2142 hospitals. Median proportion of hospitals reporting payer-negotiated rates was 18.3% (interquartile range [IQR]:17.2%-18.8%) for panel-based and 30.3% (IQR:22.2%-37.8%) for non-panel-based tests; median negotiated rates per test were $3704 (IQR: $3096-$4129) and $301 (IQR: $157-$485), respectively. Negotiated rates varied within hospitals (median within-hospital ratios: panel-based tests, 1.5 [IQR:1.4-1.5]; non-panel-based tests, 1.6 [IQR: 1.5-1.7]) and between hospitals (median between-hospital ratios: panel-based tests, 5.5 [IQR: 5.5-5.6]; non-panel-based tests, 5.2 [IQR: 5.1-5.4]). Negotiated rates varied significantly by payer for all tests.

**Conclusion:**

Our findings revealed wide variation in commercial payer-negotiated rates for oncology biomarker tests, underscoring the need for pricing transparency and efforts to reduce price variation to support equitable, affordable cancer care.

## Introduction

Precision oncology testing is the use of clinically validated molecular tests to identify biomarkers that enable personalized cancer care.^1,2^ These tests range from targeted mutation assays to comprehensive genomic panels, detecting hundreds of genomic alterations simultaneously, and are commonly used to guide prognosis and therapy selection.^1,3,4^ Biomarker-directed therapies, shown to improve clinical outcomes compared with non-biomarker-informed approaches in several solid tumors, including breast, colon, and non-small cell lung cancer,^5-8^ are increasingly incorporated into clinical practice guidelines,^9-12^ professional society recommendations,^13^ and regulatory decision-making.^14^ For instance, the National Comprehensive Cancer Network (NCCN) Biomarkers Compendium includes over 1700 biomarker testing recommendations that inform clinical and coverage decisions in the United States,^15^ the European Society for Medical Oncology (ESMO) has established evidence-based frameworks to guide interpretation of genomic alterations and treatment selection (eg, the ESMO Scale for Clinical Actionability of Molecular Targets [ESCAT]),^16,17^ and the US Food and Drug Administration (FDA) incorporates biomarker testing into oncology drug approvals.^14,18^

However, as biomarker testing has become integral to oncology care and a prerequisite for access to many guideline-recommended therapies, concerns have emerged over its impact on cancer care costs.^3,19^ Precision oncology tests commonly cost several hundred to several thousand US dollars per test, representing a potential financial burden beyond the already high costs of cancer care for both health systems and patients.^3,19-23^ Insurance coverage for these tests remains inconsistent,^24^ with broad next-generation sequencing panels frequently subject to coverage denials or partial reimbursement.^3,25^ Although policy protections limit patient liability for unexpected laboratory charges,^26^ patient cost sharing may be substantial and may influence treatment decisions, leading some patients to forgo clinically indicated testing.^3,19,27-29^ Beyond patient-facing cost and coverage issues, little is known about the prices that hospitals negotiate with commercial payers for precision oncology tests and the extent to which these prices vary across different settings, which can have important implications for health systems, payers, and patients.

In January 2021, the Centers for Medicare & Medicaid Services (CMS) implemented the Hospital Price Transparency Rule, requiring hospitals to publicly report payer-negotiated prices for selected services.^30^ Prior analyses of these data have demonstrated substantial variation in negotiated prices for a wide range of health care services, including surgical procedures, diagnostic imaging, and specialty care across multiple disciplines.^31-36^ In this study, we evaluated commercial payer-negotiated rates for precision oncology tests that play a major role in cancer treatment decisions, including breast cancer, non-small cell lung cancer, and colon cancer,^37^ and examined variation in rates within and between hospitals, as well as differences across payers and geographic location.

## Materials and methods

### Study design and data sources

We conducted a cross-sectional study using hospital price data from Turquoise Health, which aggregates prices reported by US hospitals in accordance with the CMS Hospital Price Transparency Rule. Turquoise Health standardizes heterogeneous hospital machine-readable files into a common analytic structure with validation workflows and traceability to underlying source files and calculations.^38-40^ We followed the Strengthening the Reporting of Observational Studies in Epidemiology (STROBE) guideline. Because this study uses non-patient-level data, institutional review board approval and informed consent were not required.

### Sample construction

We focused on biomarker tests commonly used to inform decision-making for 3 high-burden cancers: breast, non-small cell lung, and colon cancer.^9-11,37,41^ For each cancer type, we identified eligible biomarker tests using the NCCN Biomarkers Compendium,^15^ consistent with the broad definition of precision oncology as the use of biomarker testing to individualize patient care, including diagnosis, prognosis, therapy selection, and treatment monitoring,^1,2,42^ We then mapped them to the corresponding CMS-defined Current Procedural Terminology (CPT) codes.^43^ Tests were excluded if they lacked a unique CPT code (ie, codes shared across several tests) or if the codes corresponded to a panel not explicitly recommended in the NCCN Compendium; only codes directly corresponding to the specified biomarker tests were retained. For example, when the NCCN Compendium recommended testing for a specific gene (eg, *KRAS*), we included only the CPT code(s) corresponding to that gene and excluded CPT codes for broader panels containing it. In contrast, when the NCCN Compendium recommended a defined panel (eg, Oncotype DX Breast Recurrence Score), we included the CPT code(s) corresponding to that panel. Lastly, for biomarker tests with more than one CPT code, we included only the most comprehensive code. When no single CPT code was clearly the most comprehensive, we included the code with the greatest number of reported rates in Turquoise Health database.

To capture panels used in practice but not explicitly recommended by NCCN guidelines, we reviewed the CMS Clinical Laboratory Fee Schedule to identify CPT codes corresponding to oncology panel tests, including multigene DNA and RNA assays and protein-based tests. From these, we selected tests used for clinical decision-making in breast, non-small cell lung, or colon cancer, as well as pan-solid tumor panel tests used in the management of these 3 cancer types. To ensure comparability across tests, inclusion was limited to CPT codes mapping to a single, uniquely defined assay with a fixed number of genes or analytes. We then limited the sample to the most frequently reported panels, each with more than 10 000 reported negotiated rates, in Turquoise Health database.

### Data extraction and curation

We searched the Turquoise Health database for each CPT code to identify rates negotiated by the four largest US private payers (Aetna, Blue Cross Blue Shield, Cigna, and UnitedHealthcare, which together represent approximately 80% of the US commercial insurance market),^44^ as of January 2026. We limited the analysis to outpatient or combined inpatient and outpatient fee schedule rates reported by short-term acute care hospitals, excluding critical access (a federal designation for small, rural hospitals intended to preserve access to care in underserved areas), children's, rehabilitation, psychiatric, long-term acute care, and rural emergency hospitals. We included only facility fees and excluded professional fees and rates containing modifiers.

Next, we excluded non-standard coding formats and rates outside the 1st and 99th percentiles for each biomarker test to reduce outlier effects.^35^ We adjusted all rates by Geographic Adjustment Factor (GAF) at the ZIP code level to account for geographic cost differences.^34^ Hospital rurality was determined by linking hospital ZIP codes to counties using a ZIP code-to-county crosswalk, and classifying counties according to the National Center for Health Statistics (NCHS) Urban-Rural Classification Scheme,^45^ with codes 1-4 categorized as urban and codes 5-6 categorized as rural.^46^

### Statistical analysis

We calculated the proportion of hospitals reporting at least one negotiated rate for biomarker tests in the sample among all US acute care hospitals in the Turquoise Health database. For each hospital, we calculated the median adjusted commercial payer-negotiated rate for each test, along with the interquartile range (IQR) and the 10th and 90th percentiles. We calculated within-hospital price variation as the ratio of the 90th-to-10th percentile of negotiated rates for each hospital, and between-hospital variation as the ratio of the 90th-to-10th percentile of median negotiated rates across hospitals. These percentile-based measures mitigate the influence of outliers, consistent with prior price variation research.^47^ We stratified rates by payer (Aetna, Blue Cross Blue Shield, Cigna, UnitedHealthcare), hospital size (<100, 100-499, 500-999, ≥1000 beds), and rurality. Differences by payer, hospital size, and rurality were evaluated using Generalized Estimating Equation (GEE) linear models with robust standard errors to account for clustering of repeated negotiated rates within hospital-payer combinations. Overall Wald tests were used to assess statistical significance. The Benjamini–Hochberg false discovery rate (FDR) procedure was applied to account for multiple comparisons across biomarker-specific analyses. Analyses were conducted using RStudio (version 2023.09.1 + 494, Posit), with two-tailed *P* < 0.05 considered statistically significant.

### Limitations

This study has several limitations. First, not all biomarker tests used in oncology practice were included; however, the tests examined span a range of testing modalities, clinical indications, and price levels, and are used in settings where precision oncology plays a central role in treatment decision-making. Second, the analysis was limited to hospitals included in the Turquoise Health database, which may not capture all US hospitals that perform or bill for oncology biomarker testing; additionally, hospitals in Maryland were excluded because prices in that state are set through an all-payer rate-setting system administered by the Maryland Health Services Cost Review Commission.^48^ Third, negotiated rates were derived from hospital-reported price transparency files, and data completeness and accuracy may vary across institutions despite Turquoise Health's parsing, validation, and outlier-detection steps and our data cleaning and exclusion of extreme outliers.

## Results

### Sample construction

We identified 41 biomarker tests from the NCCN Biomarkers Compendium for breast, colon, or non-small cell lung cancer and 37 oncology panel tests used for clinical decision-making in these 3 cancers, corresponding to a single, uniquely defined assay with a fixed number of genes or analytes, in the CMS Clinical Laboratory Fee Schedule files (Appendix Table S1). Of the 41 biomarker tests identified from the NCCN Biomarkers Compendium, 1 (2.4%) unspecified test listed as “germline mutation testing” for breast cancer risk assessment and 19 (46.3%) tests without unique CPT codes were excluded (Appendix Tables S2 and S3). Two biomarker tests (*CA15–3* and *CA27.29*) shared a single CPT code, resulting in 20 unique CPT-coded biomarker tests recommended by the NCCN Biomarkers Compendium (Appendix Figure S1). Of the 37 panel tests identified from the CMS Clinical Laboratory Fee Schedule files, 8 (21.6%) had more than 10 000 reported commercial payer-negotiated rates in the Turquoise Health database. The final merged sample included 28 biomarker tests, comprising 20 tests from the NCCN Biomarkers Compendium and 8 oncology panel tests from the CMS Clinical Laboratory Fee Schedule files.

### Sample characteristics

Among the 28 biomarker tests, 13 (46.4%) were panel-based, commercially developed tests often performed as send-out tests by centralized laboratories: 6 for breast cancer (Breast Cancer Index, EndoPredict, MammaPrint, Oncotype DX Breast Recurrence Score, Oncotype DX DCIS Score, and Prosigna), 4 for breast, colon, and non-small cell lung cancer (FoundationOne, MSK-IMPACT, Oncotype MAP PanCancer Tissue Test, and PGDx elio Tissue Complete), 2 for non-small cell lung cancer (Oncomine Dx Target test and VeriStrat), and 1 for colon cancer management (Oncotype DX Colon Cancer Assay). The remaining 15 (53.6%) were non-panel-based tests assessing one or more molecular targets but not marketed or ordered as panels: 5 for breast cancer (*BRCA1/2* mutation, *CA15–3* or *CA27.29* expression, estrogen receptor expression, *PALB2* mutation, and progesterone receptor expression testing), 4 for breast and colon cancer (*CEA* expression, mismatch repair, microsatellite instability, and *PIK3CA* mutation testing), 2 for colon cancer (*DPYD* variant and *KRAS/NRAS* mutation testing), 2 for non-small cell lung cancer (*EGFR* mutation and *KRAS* mutation testing), 1 for breast and non-small cell lung cancer (*NTRK* gene fusion testing), and 1 for colon and non-small cell lung cancer management (*BRAF* mutation testing).

### Payer-negotiated price reporting

A total of 422 515 payer-negotiated rates were identified for the 28 biomarker tests, with a median of 10 906 (IQR, 9719–17 598) rates per biomarker test and a median of 6 (IQR, 6-6) rates per hospital per biomarker test (Table 1). At least one payer-negotiated rate for an included biomarker test was reported by 2142 acute care hospitals, representing 55.3% of the 3874 acute care hospitals in the Turquoise Health database, across 49 US states and the District of Columbia.

**Table 1.** Price reporting availability for biomarker tests used in precision oncology .

Biomarker test (CPT code)	Indication	Median (IQR) number of negotiated rates per hospital^a^	Number of commercial payer-negotiated rates^b^	Number (%) of hospitals in the Turquoise Health database reporting negotiated rate^c^, (*n* = 3874)
***Panel-based biomarker tests***				
Breast Cancer Index, 11-gene (81518)	Breast Cancer	6 (3-11)	10 614	713 (18.4%)
EndoPredict, 12-gene (81522)	Breast Cancer	5 (3-10)	8074	705 (18.2%)
FoundationOne, 324-gene (0037U)	Several cancer types, including breast, colon, and non-small cell lung cancer	6 (2-11)	11 610	803 (20.7%)
MammaPrint, 70-gene (81521)	Breast cancer	6 (3-11)	10 428	727 (18.8%)
MSK-IMPACT, 468-gene (0048U)	Several cancer types, including breast, colon, and non-small cell lung cancer	5 (2-11)	9480	646 (16.7%)
Oncotype DX Breast Recurrence Score, 21-gene (81519)	Breast cancer	6 (3-12)	12 154	838 (21.6%)
Oncotype DX Colon Cancer Assay, 12-gene (81525)	Colon cancer	6 (3-11)	10 184	717 (18.5%)
Oncotype DX DCIS Score, 12-gene (0045U)	Breast cancer	6 (2-11)	9970	654 (16.9%)
Oncotype MAP PanCancer Tissue Test, 257-gene (0244U)	Several cancer types, including breast, colon, and non-small cell lung cancer	6 (2-11)	9472	666 (17.2%)
Oncomine™ Dx Target Test, 23-gene (0022U)	Non-small cell lung cancer	5 (2-11)	9214	638 (16.5%)
PGDx elio™ Tissue Complete, 505-gene (0250U)	Several cancer types, including breast, colon, and non-small cell lung cancer	6 (2-11)	9583	685 (17.7%)
Prosigna, 50-gene (81520)	Breast cancer	6 (3-11)	11 197	751 (19.4%)
VeriStrat, 8-protein (81538)	Non-small cell lung cancer	6 (3-12)	10 158	708 (18.3%)
***Non-panel-based biomarker tests***				
*BRAF* mutation (81210)	Non-small cell lung cancer and colon cancer	8 (3-18)	31 306	1537 (39.7%)
*BRCA1/2* mutation (81162)	Breast cancer	6 (3-12)	18 827	1285 (33.2%)
*CA15–3* or *CA27.29* expression (86300)	Breast cancer	10 (4-21)	39 409	1869 (48.2%)
*CEA* expression (82378)	Breast cancer and colon cancer	8 (4-20)	40 571	1915 (49.4%)
*DPYD* variant testing (81232)	Colon cancer	6 (3-11)	17 188	1172 (30.3%)
*EGFR* mutation (81235)	Non-small cell lung cancer	7 (3-14)	23 896	1494 (38.6%)
Estrogen receptor expression (84233)	Breast cancer	6 (3-12)	12 322	893 (23.1%)
*KRAS* mutation (81275)	Non-small cell lung cancer	7 (3-16)	24 019	1435 (37.0%)
*KRAS/NRAS* mutation (0111U)	Colon cancer	6 (3-11)	9764	656 (16.9%)
Mismatch repair testing (0160U)	Breast cancer and colon cancer	6 (2-11)	8980	610 (15.7%)
Microsatellite instability testing (81301)	Breast cancer and colon cancer	7 (3-13)	21 257	1342 (34.6%)
*NTRK* gene fusion testing (81194)	Non-small cell lung cancer and breast cancer	6 (3-11)	11 668	888 (22.9%)
*PALB2* mutation (81307)	Breast cancer	5 (3-10)	9300	831 (21.5%)
*PIK3CA* mutation (81309)	Breast cancer and colon cancer	5 (3-11)	10 116	905 (23.4%)
Progesterone receptor expression (84234)	Breast cancer	7 (3-12)	11 754	830 (21.4%)

Source: Authors' analysis of data from the Turquoise Health hospital pricing database.

^a^Median (IQR) number of negotiated rates per hospital refers to the number of distinct commercial payer–negotiated rates reported per hospital for each biomarker test.

^b^Number of commercial payer-negotiated rates indicates the total number of distinct payer-negotiated rates identified for each biomarker test across all included hospitals and payers.

^c^Number (%) of hospitals reporting a negotiated rate refers to the count and proportion of hospitals with at least one reported commercial payer-negotiated rate for each biomarker test.

Abbreviations: CPT, current procedural terminology; IQR, interquartile range.

The median proportion of hospitals reporting a payer-negotiated rate was 18.3% (IQR, 17.2%-18.8%) for panel-based tests and 30.3% (IQR, 22.2%-37.8%) for non-panel-based tests. Among panel-based tests, reporting was highest for Oncotype DX Breast Recurrence Score for breast cancer (21.6%) and lowest for Oncomine Dx for non-small cell lung cancer (16.5%). Among non-panel-based tests, reporting was highest for *CEA* expression for breast and colon cancer (49.4%) and lowest for mismatch repair testing for the same cancers (15.7%).

### Payer-negotiated rates

Median negotiated rates per test were $3704 (IQR, $3096-$4129) for panel-based and $301 (IQR, $157-$485) for non-panel-based tests. Among panel-based tests, the highest median rate was for EndoPredict for breast cancer ($4877 [IQR, $3886-$15 898]) and the lowest for Oncomine Dx for non-small cell lung cancer ($1982 [IQR, $1802-$2210]) (Table 2). Among non-panel-based tests, the highest median rate was for *BRCA1/2* mutation testing for breast cancer ($2241 [IQR, $1877-$5001]) and the lowest for *CEA* expression testing for breast and colon cancer ($28 [IQR, $20-$59]).

**Table 2.** Price variability for biomarker tests used in precision oncology .

Biomarker test (CPT code)	Negotiated rates, USD, median (IQR)	Within-hospital 90th:10th percentile negotiated rate ratio^a^, median (IQR)	Between-hospital 90th:10th percentile negotiated rate ratio^b^
***Panel-based biomarker tests***			
Breast Cancer Index, 11-gene (81518)	$4150 (3873–11 380)	1.4 (1.0-2.8)	5.2
EndoPredict, 12-gene (81522)	$4877 (3886–15 898)	1.5 (1.0-3.1)	6.6
FoundationOne, 324-gene (0037U)	$3751 (3521–11 826)	1.5 (1.0-2.6)	5.5
MammaPrint, 70-gene (81521)	$4150 (3873–15 528)	1.5 (1.0-2.8)	5.8
MSK-IMPACT, 468-gene (0048U)	$3096 (2920-9944)	1.3 (1.0-2.5)	5.0
Oncotype DX Breast Recurrence Score, 21-gene (81519)	$4129 (3869–10 680)	1.6 (1.0-2.8)	5.5
Oncotype DX Colon Cancer Assay, 12-gene (81525)	$3304 (3113-9688)	1.5 (1.0-2.8)	5.3
Oncotype DX DCIS Score, 12-gene (0045U)	$4120 (3896–15 528)	1.4 (1.0-2.8)	5.5
Oncotype MAP PanCancer Tissue Test, 257-gene (0244U)	$3704 (3500–13 899)	1.4 (1.0-2.6)	5.6
Oncomine™ Dx Target Test, 23-gene (0022U)	$1982 (1802-2210)	1.2 (1.0-3.2)	33.7
PGDx elio™ Tissue Complete, 505-gene (0250U)	$3113 (2920–11 706)	1.3 (1.0-2.5)	5.6
Prosigna, 50-gene (81520)	$2690 (2525-9635)	1.5 (1.0-3.0)	5.6
VeriStrat, 8-protein (81538)	$3045 (2868-9232)	1.6 (1.0-3.1)	5.5
***Non-panel-based biomarker tests***			
*BRAF* mutation (81210)	$223 (180-483)	1.7 (1.0-3.0)	5.2
*BRCA1/2* mutation (81162)	$2241 (1877-5001)	1.5 (1.0-2.9)	4.7
*CA15-3* or *CA27.29* expression (86300)	$32 (22-65)	1.9 (1.1-3.5)	5.0
*CEA* expression (82378)	$28 (20-59)	1.9 (1.1-3.5)	5.1
*DPYD* variant testing (81232)	$220 (180-484)	1.5 (1.0-2.7)	4.7
*EGFR* mutation (81235)	$413 (332-895)	1.7 (1.0-3.0)	5.1
Estrogen receptor expression (84233)	$95 (88-294)	1.7 (1.0-3.1)	5.5
*KRAS* mutation (81275)	$246 (199-535)	1.7 (1.0-2.9)	5.1
*KRAS/NRAS* mutation (0111U)	$724 (686-2599)	1.3 (1.0-2.5)	5.3
Mismatch repair testing (0160U)	$301 (283-1134)	1.3 (1.0-3.0)	5.4
Microsatellite instability testing (81301)	$415 (359-962)	1.6 (1.0-2.9)	4.7
*NTRK* gene fusion testing (81194)	$555 (518-1883)	1.5 (1.0-2.8)	5.4
*PALB2* mutation (81307)	$725 (626-1775)	1.6 (1.0-3.2)	10.1
*PIK3CA* mutation (81309)	$350 (277-1102)	1.6 (1.0-3.0)	6.6
Progesterone receptor expression (84234)	$74 (65-254)	1.7 (1.0-3.2)	5.4

Source: Authors' analysis of data from the Turquoise Health hospital pricing database.

All commercial payer-negotiated rates are normalized using the Geographic Adjustment Factor (GAF) to account for geographic cost differences and reported in 2026 USD. Excludes commercial payer-negotiated rates below the 1st percentile and above the 99th percentile for each CPT code.

^a^Calculated at the hospital-level as the ratio of 90th-to-10th percentile commercial payer-negotiated rates.

^b^Calculated as the ratio of 90th-to-10th percentile commercial payer-negotiated rate, across all reported commercial payer-negotiated rates.

Abbreviations: CPT, Current Procedural Terminology; IQR, interquartile range; USD, US dollar.

### Within- and between-hospital payer-negotiated rate variation

Among panel-based tests, the median within-hospital ratio of 90th-to-10th percentile rates ranged from 1.2 (Oncomine Dx for non-small cell lung cancer) to 1.6 (VeriStrat for non-small cell lung cancer), with a median of 1.5 (IQR, 1.4-1.5). Between-hospital ratios ranged from 5.0 (MSK-IMPACT for breast, colon, and non-small cell lung cancer) to 33.7 (Oncomine Dx for non-small cell lung cancer), with a median of 5.5 (IQR, 5.5-5.6).

Among non-panel-based tests, the median within-hospital ratio of 90th-to-10th percentile rates ranged from 1.3 (*KRAS/NRAS* mutation for colon cancer and mismatch repair testing for breast and colon cancer) to 1.9 (*CEA* expression for breast and colon cancer and *CA15–3* or *CA27.29* expression for breast cancer), with a median of 1.6 (IQR, 1.5-1.7). Between-hospital ratios ranged from 4.7 (*BRCA1/2* mutation testing for breast cancer, *DPYD* variant testing for colon cancer, and microsatellite instability testing for breast and colon cancer) to 10.1 (*PALB2* mutation testing for breast cancer), with a median of 5.2 (IQR, 5.1-5.4).

### Payer-negotiated rate variation by payer

There were statistically significant differences in mean negotiated rates across payers for all panel- and non-panel-based biomarker tests after adjustment for multiple comparisons using the FDR procedure (all FDR-adjusted *P* < 0.05; Table 3). Among panel-based tests, Blue Cross Blue Shield had the highest mean payer-negotiated rate for 6 tests (46.2%), Cigna for 4 tests (30.8%), and Aetna for 3 tests (23.1%). UnitedHealthcare had the lowest payer-negotiated rate for all 13 tests (100.0%). Among non-panel-based tests, Cigna had the highest mean negotiated rate for 8 tests (53.3%), Blue Cross Blue Shield for 4 tests (26.7%), and Aetna for 3 tests (20.0%); UnitedHealthcare had the lowest negotiated rate for all 15 tests (100.0%).

**Table 3.** Price variability of biomarker tests used in precision oncology by payer.

Biomarker test (CPT code)	Payer name	Mean difference, USD (95% CI)	*P* value	Overall *P* value	FDR-adjusted Overall *P* value
***Panel-based biomarker tests***					
Breast Cancer Index (81518)	Aetna	Reference	Not applicable	<0.001	<0.001
	Blue Cross Blue Shield	$−702 (−1501 to 97)	0.09		
	Cigna	$−307 (−1206 to 591)	0.50		
	UnitedHealthcare	$−4354 (−5034 to −3673)	<0.001		
EndoPredict, 12 gene (81522)	Aetna	Reference	Not applicable	<0.001	<0.001
	Blue Cross Blue Shield	$−788 (−1726 to 151)	0.1		
	Cigna	$−1006 (−1968 to −43)	0.04		
	UnitedHealthcare	$−4869 (−5616 to −4122)	<0.001		
FoundationOne, 324-gene (0037U)	Aetna	Reference	Not applicable	<0.001	<0.001
	Blue Cross Blue Shield	$1166 (447 to 1885)	0.001		
	Cigna	$878 (104 to 1652)	0.03		
	UnitedHealthcare	$−3525 (−4067 to −2983)	<0.001		
MammaPrint, 70-gene (81521)	Aetna	Reference	Not applicable	<0.001	<0.001
	Blue Cross Blue Shield	$−267 (−1124 to 590)	0.54		
	Cigna	$−276 (−1201 to 649)	0.56		
	UnitedHealthcare	$−4265 (−4963 to −3566)	<0.001		
MSK-IMPACT, 468-gene (0048U)	Aetna	Reference	Not applicable	<0.001	<0.001
	Blue Cross Blue Shield	$95 (−590 to 780)	0.8		
	Cigna	$86 (−656 to 828)	0.8		
	UnitedHealthcare	$−3054 (−3629 to −2478)	<0.001		
Oncotype DX Breast Recurrence Score, 21-gene (81519)	Aetna	Reference	Not applicable	<0.001	<0.001
	Blue Cross Blue Shield	$−833 (−1563 to −102)	0.03		
	Cigna	$516 (−295 to 1327)	0.21		
	UnitedHealthcare	$−4192 (−4807 to −3577)	<0.001		
Oncotype DX Colon Cancer Assay, 12-gene (81525)	Aetna	Reference	Not applicable	<0.001	<0.001
	Blue Cross Blue Shield	$139 (−523 to 800)	0.68		
	Cigna	$700 (−2 to 1403)	0.05		
	UnitedHealthcare	$−2919 (−3457 to −2380)	<0.001		
Oncotype DX DCIS Score, 12-gene (0045U)	Aetna	Reference	Not applicable	<0.001	<0.001
	Blue Cross Blue Shield	$446 (−490 to 1381)	0.4		
	Cigna	$146 (−841 to 1134)	0.8		
	UnitedHealthcare	$−4091 (−4859 to −3324)	<0.001		
Oncotype MAP PanCancer Tissue Test, 257-gene (0244U)	Aetna	Reference	Not applicable	<0.001	<0.001
	Blue Cross Blue Shield	$286 (−570 to 1143)	0.51		
	Cigna	$934 (−59 to 1926)	0.07		
	UnitedHealthcare	$−3146 (−3860 to −2431)	<0.001		
Oncomine™ Dx Target Test, 23-gene (0022U)	Aetna	Reference	Not applicable	<0.001	<0.001
	Blue Cross Blue Shield	$593 (127 to 1059)	0.01		
	Cigna	$−395 (−894 to 104)	0.12		
	UnitedHealthcare	$−1312 (−1702 to −921)	<0.001		
PGDx elio™ Tissue Complete, 505-gene (0250U)	Aetna	Reference	Not applicable	<0.001	<0.001
	Blue Cross Blue Shield	$976 (208 to 1744)	0.01		
	Cigna	$274 (−559 to 1107)	0.52		
	UnitedHealthcare	$−3017 (−3621 to −2414)	<0.001		
Prosigna, 50-gene (81520)	Aetna	Reference	Not applicable	<0.001	<0.001
	Blue Cross Blue Shield	$213 (−357 to 783)	0.46		
	Cigna	$−407 (−989 to 176)	0.17		
	UnitedHealthcare	$−2892 (−3344 to −2440)	<0.001		
VeriStrat, 8-protein (81538)	Aetna	Reference	Not applicable	<0.001	<0.001
	Blue Cross Blue Shield	$52 (−555 to 660)	0.87		
	Cigna	$455 (−169 to 1078)	0.15		
	UnitedHealthcare	$−2747 (−3223 to −2271)	<0.001		
***Non-panel-based biomarker tests***					
*BRAF* mutation (81210)	Aetna	Reference	Not applicable	<0.001	<0.001
	Blue Cross Blue Shield	$−1 (−25 to 23)	0.933		
	Cigna	$23 (−4 to 50)	0.093		
	UnitedHealthcare	$−177 (−197 to −158)	<0.001		
*BRCA1/2* mutation (81162)	Aetna	Reference	Not applicable	<0.001	<0.001
	Blue Cross Blue Shield	$163 (−129 to 455)	0.275		
	Cigna	$266 (−51 to 583)	0.1		
	UnitedHealthcare	$−1892 (−2130 to −1655)	<0.001		
*CA15-3* or *CA27.29* expression (86300)	Aetna	Reference	Not applicable	<0.001	<0.001
	Blue Cross Blue Shield	$1 (−2 to 4)	0.474		
	Cigna	$−1 (−4 to 3)	0.616		
	UnitedHealthcare	$−25 (−28 to −22)	<0.001		
*CEA* expression (82378)	Aetna	Reference	Not applicable	<0.001	<0.001
	Blue Cross Blue Shield	$−0 (−3 to 3)	0.904		
	Cigna	$−3 (−6 to 1)	0.13		
	UnitedHealthcare	$−26 (−29 to −23)	<0.001		
*DPYD* variant testing (81232)	Aetna	Reference	Not applicable	<0.001	<0.001
	Blue Cross Blue Shield	$−27 (−57 to 2)	0.067		
	Cigna	$−18 (−51 to 14)	0.27		
	UnitedHealthcare	$−204 (−228 to −180)	<0.001		
*EGFR* mutation (81235)	Aetna	Reference	Not applicable	<0.001	<0.001
	Blue Cross Blue Shield	$−62 (−105 to −19)	0.005		
	Cigna	$3 (−46 to 51)	0.917		
	UnitedHealthcare	$−340 (−375 to −304)	<0.001		
Estrogen receptor expression (84233)	Aetna	Reference	Not applicable	<0.001	<0.001
	Blue Cross Blue Shield	$10 (−7 to 27)	0.25		
	Cigna	$19 (0 to 37)	0.045		
	UnitedHealthcare	$−92 (−106 to −78)	<0.001		
*KRAS* mutation (81275)	Aetna	Reference	Not applicable	<0.001	<0.001
	Blue Cross Blue Shield	$−22 (−49 to 5)	0.10		
	Cigna	$4 (−25 to 34)	0.77		
	UnitedHealthcare	$−215 (−237 to −193)	<0.001		
*KRAS/NRAS* mutation (0111U)	Aetna	Reference	Not applicable	<0.001	<0.001
	Blue Cross Blue Shield	$177 (16 to 337)	0.03		
	Cigna	$129 (−42 to 301)	0.14		
	UnitedHealthcare	$−602 (−733 to −470)	<0.001		
Mismatch repair testing (0160U)	Aetna	Reference	Not applicable	<0.001	<0.001
	Blue Cross Blue Shield	$223 (134 to 311)	<0.001		
	Cigna	$136 (51 to 220)	0.002		
	UnitedHealthcare	$−299 (−355 to −242)	<0.001		
Microsatellite instability testing (81301)	Aetna	Reference	Not applicable	<0.001	<0.001
	Blue Cross Blue Shield	$−54 (−103 to −5)	0.03		
	Cigna	$15 (−40 to 71)	0.58		
	UnitedHealthcare	$−380 (−420 to −339)	<0.001		
*NTRK* gene fusion testing (81194)	Aetna	Reference	Not applicable	<0.001	<0.001
	Blue Cross Blue Shield	$5 (−100 to 109)	0.93		
	Cigna	$37 (−87 to 160)	0.56		
	UnitedHealthcare	$−487 (−576 to −399)	<0.001		
*PALB2* mutation (81307)	Aetna	Reference	Not applicable	<0.001	<0.001
	Blue Cross Blue Shield	$−392 (−520 to −264)	<0.001		
	Cigna	$109 (−54 to 273)	0.19		
	UnitedHealthcare	$−731 (−848 to −613)	<0.001		
*PIK3CA* mutation (81309)	Aetna	Reference	Not applicable	<0.001	<0.001
	Blue Cross Blue Shield	$−56 (−112 to 1)	0.05		
	Cigna	$−34 (−95 to 28)	0.28		
	UnitedHealthcare	$−337 (−383 to −291)	<0.001		
Progesterone receptor expression (84234)	Aetna	Reference	Not applicable	<0.001	<0.001
	Blue Cross Blue Shield	$16 (2 to 31)	0.03		
	Cigna	$16 (1 to 32)	0.04		
	UnitedHealthcare	$−73 (−84 to −61)	<0.001		

Source: Authors' analysis of data from the Turquoise Health hospital pricing database.

Abbreviations: CPT, current procedural terminology; FDR, false discovery rate; IQR, interquartile range; USD, US dollar.

### Payer-negotiated rate variation by hospital size

Differences in mean negotiated rates across hospital size categories were statistically significant for 4 of 13 panel-based tests (30.8%) and 4 of 15 non-panel-based tests (26.7%) without adjustment for multiple comparisons; however, none remained significant after FDR adjustment (Table 4). Mean negotiated rates were higher in hospitals with 1000 or more beds than in each of the 3 smaller categories (<100, 100-499, and 500-999 beds) for all 13 panel-based tests (100.0%) and for 7 of 15 non-panel-based tests (46.7%).

**Table 4.** Price variability of biomarker tests used in precision oncology based on hospital size .

Biomarker test (CPT code)	Hospital size (based on number of beds)	Mean difference, USD (95% CI)	*P* value	Overall *P* value	FDR-adjusted overall *P* value
***Panel-based biomarker tests***					
Breast Cancer Index (81518)	<100	Reference	Not applicable	0.10	0.18
	100-499	$−175 (−757 to 408)	0.56		
	500-999	$−149 (−1069 to 771)	0.75		
	≥1000	$1544 (154 to 2933)	0.03		
EndoPredict, 12 gene (81522)	<100	Reference	Not applicable	0.15	0.20
	100-499	$59 (−587 to 705)	0.86		
	500-999	$−153 (−1154 to 848)	0.76		
	≥1000	$1650 (164 to 3135)	0.03		
FoundationOne, 324-gene (0037U)	<100	Reference	Not applicable	0.76	0.76
	100-499	$−158 (−698 to 382)	0.57		
	500-999	$160 (−646 to 966)	0.70		
	≥1000	$281 (−1065 to 1627)	0.68		
MammaPrint, 70-gene (81521)	<100	Reference	Not applicable	0.17	0.20
	100-499	$−53 (−663 to 557)	0.87		
	500-999	$−174 (−1144 to 797)	0.73		
	≥1000	$1533 (79 to 2987)	0.04		
MSK-IMPACT, 468-gene (0048U)	<100	Reference	Not applicable	0.10	0.18
	100-499	$−56 (−535 to 422)	0.82		
	500-999	$−76 (−819 to 668)	0.84		
	≥1000	$1378 (216 to 2540)	0.02		
Oncotype DX Breast Recurrence Score, 21-gene (81519)	<100	Reference	Not applicable	0.02	0.16
	100-499	$−334 (−874 to 205)	0.23		
	500-999	$−275 (−1139 to 588)	0.53		
	≥1000	$1615 (342 to 2888)	0.01		
Oncotype DX Colon Cancer Assay, 12-gene (81525)	<100	Reference	Not applicable	0.04	0.16
	100-499	$−283 (−759 to 192)	0.24		
	500-999	$−160 (−949 to 629)	0.69		
	≥1000	$1308 (128 to 2488)	0.03		
Oncotype DX DCIS Score, 12-gene (0045U)	<100	Reference	Not applicable	0.12	0.18
	100-499	$−17 (−664 to 630)	0.96		
	500-999	$−162 (−1178 to 854)	0.76		
	≥1000	$1744 (221 to 3268)	0.03		
Oncotype MAP PanCancer Tissue Test, 257-gene (0244U)	<100	Reference	Not applicable	0.049	0.16
	100-499	$−118 (−717 to 480)	0.70		
	500-999	$−181 (−1105 to 743)	0.7		
	≥1000	$1903 (437 to 3368)	0.01		
Oncomine™ Dx Target Test, 23-gene (0022U)	<100	Reference	Not applicable	0.03	0.16
	100-499	$−32 (−331 to 267)	0.834		
	500-999	$−192 (−643 to 260)	0.406		
	≥1000	$1105 (301 to 1909)	0.007		
PGDx elio™ Tissue Complete, 505-gene (0250U)	<100	Reference	Not applicable	0.10	0.18
	100-499	$−240 (−789 to 309)	0.39		
	500-999	$−134 (−1010 to 743)	0.77		
	≥1000	$1253 (24 to 2482)	0.046		
Prosigna, 50-gene (81520)	<100	Reference	Not applicable	0.12	0.18
	100-499	$−89 (−500 to 323)	0.67		
	500-999	$−39 (−679 to 602)	0.91		
	≥1000	$989 (80 to 1898)	0.03		
VeriStrat, 8-protein (81538)	<100	Reference	Not applicable	0.22	0.24
	100-499	$−110 (−555 to 336)	0.63		
	500-999	$−23 (−758 to 712)	0.95		
	≥1000	$1005 (−75 to 2086)	0.07		
***Non-panel-based biomarker tests***					
*BRAF* mutation (81210)	<100	Reference	Not applicable	0.04	0.13
	100-499	$−24 (−43 to −4)	0.017		
	500-999	$2 (−27 to 32)	0.881		
	≥1000	$−8 (−53 to 36)	0.715		
*BRCA1/2* mutation (81162)	<100	Reference	Not applicable	0.61	0.73
	100-499	$−120 (−341 to 101)	0.289		
	500-999	$25 (−325 to 375)	0.889		
	≥1000	$33 (−453 to 518)	0.895		
*CA15-3* or *CA27.29* expression (86300)	<100	Reference	Not applicable	0.64	0.73
	100-499	$−1 (−4 to 1)	0.258		
	500-999	$0 (−4 to 4)	0.999		
	≥1000	$−2 (−7 to 4)	0.552		
*CEA* expression (82378)	<100	Reference	Not applicable	0.04	0.13
	100-499	$−1 (−3 to 2)	0.686		
	500-999	$−1 (−5 to 2)	0.473		
	≥1000	$−6 (−11 to −2)	0.006		
*DPYD* variant testing (81232)	<100	Reference	Not applicable	0.76	0.76
	100-499	$−12 (−34 to 11)	0.317		
	500-999	$−5 (−38 to 27)	0.748		
	≥1000	$1 (−49 to 51)	0.968		
*EGFR* mutation (81235)	<100	Reference	Not applicable	0.007	0.11
	100-499	$−51 (−86 to −16)	0.005		
	500-999	$3 (−50 to 57)	0.907		
	≥1000	$−72 (−147 to 4)	0.063		
Estrogen receptor expression (84233)	<100	Reference	Not applicable	0.73	0.76
	100-499	$−1 (−14 to 11)	0.847		
	500-999	$5 (−16 to 26)	0.667		
	≥1000	$−13 (−39 to 14)	0.342		
*KRAS* mutation (81275)	<100	Reference	Not applicable	0.21	0.40
	100-499	$−16 (−37 to 6)	0.148		
	500-999	$12 (−21 to 45)	0.488		
	≥1000	$−15 (−65 to 36)	0.565		
*KRAS/NRAS* mutation (0111U)	<100	Reference	Not applicable	0.12	0.27
	100-499	$−14 (−126 to 98)	0.806		
	500-999	$−20 (−199 to 159)	0.827		
	≥1000	$324 (39 to 608)	0.026		
Mismatch repair testing (0160U)	<100	Reference	Not applicable	0.05	0.13
	100-499	$12 (−51 to 74)	0.712		
	500-999	$10 (−88 to 107)	0.843		
	≥1000	$182 (51 to 313)	0.006		
Microsatellite instability testing (81301)	<100	Reference	Not applicable	0.31	0.47
	100-499	$−13 (−52 to 25)	0.496		
	500-999	$35 (−25 to 95)	0.252		
	≥1000	$21 (−64 to 107)	0.623		
*NTRK* gene fusion testing (81194)	<100	Reference	Not applicable	0.04	0.13
	100-499	$−20 (−98 to 58)	0.611		
	500-999	$−19 (−131 to 93)	0.742		
	≥1000	$233 (56 to 411)	0.01		
*PALB2* mutation (81307)	<100	Reference	Not applicable	0.05	0.13
	100-499	$−16 (−116 to 84)	0.749		
	500-999	$19 (−131 to 168)	0.805		
	≥1000	$319 (74 to 564)	0.011		
*PIK3CA* mutation (81309)	<100	Reference	Not applicable	0.28	0.46
	100-499	$−14 (−56 to 28)	0.507		
	500-999	$−16 (−76 to 44)	0.608		
	≥1000	$69 (−21 to 160)	0.131		
Progesterone receptor expression (84234)	<100	Reference	Not applicable	0.42	0.57
	100-499	$3 (−7 to 14)	0.532		
	500-999	$13 (−5 to 30)	0.172		
	≥1000	$−8 (−30 to 15)	0.513		

Source: Authors' analysis of data from the Turquoise Health hospital pricing database.

Abbreviations: CPT, current procedural terminology; FDR, false discovery rate; IQR, interquartile range; USD, US dollar.

### Payer-negotiated rate variation by rurality

There were no statistically significant differences in mean negotiated rates between rural and urban hospitals for any panel-based test (Appendix Table S4**)**. Among non-panel-based tests, differences were statistically significant without adjustment for multiple comparisons for 3 of 15 (20.0%) tests, of which 2 remained statistically significant after FDR adjustment. For these tests, negotiated rates were higher in urban compared with rural hospitals.

## Discussion

This cross-sectional analysis of 28 oncology biomarker tests used in breast, colon, and non-small cell lung cancer found that median commercial payer-negotiated rates were several thousand dollars per panel-based test and several hundred dollars per non-panel-based test, with greater variation between than within hospitals. Negotiated rates also differed by payer. While more than half of acute care hospitals reported rates for at least one included test, reporting for individual tests was more limited, highlighting opportunities for improved price transparency. As oncology biomarker testing increasingly serves as a prerequisite for access to guideline-recommended therapies, variation in negotiated rates has important implications for patients, payers, and health systems.

We observed large between-hospital variations in negotiated rates for both panel-based and non-panel-based biomarker tests, with 5- to 6-fold ratios of the 90th-to-10th percentile of median negotiated rates across hospitals. This magnitude of variation is consistent with prior studies reporting wide price variation across health care services broadly and oncology products in particular.^25,49-51^ Within-hospital price variation was more modest than between-hospital variation and lower than previously reported for hospital-administered anticancer drugs. Direct comparison, however, is limited by structural differences in how biomarker tests and drugs are priced, as hospital-administered drugs are subject to payer-specific negotiation, acquisition-cost markups, and 340B Drug Pricing Program arrangements.^51-53^ This pattern of modest within-hospital but substantial between-hospital variation may suggest a greater role for hospital-level factors in negotiated rates, although specific mechanisms warrant further study.

Negotiated rates differed systematically across payers, while less consistently across hospital size and rurality. Among the four major payers, UnitedHealthcare had the lowest mean negotiated rate across all tests, which may be related to variation in payer market share and bargaining leverage.^54,55^ Higher rates observed among the largest hospitals, especially for panel-based tests, suggest that market consolidation may contribute to greater pricing leverage in negotiations with payers.^56^ Variation may also reflect factors beyond payer negotiations. Hospital price transparency files do not indicate whether testing was performed in-house or referred to an external laboratory, and billing pathways for referred laboratory services may differ across settings.^57^ Because in-house molecular testing requires specialized infrastructure, assay validation, equipment, and trained personnel, some observed variations may reflect differences in testing capacity and operational arrangements.^58^

The clinical significance of negotiated-price variation lies in its implications for patient financial burden and access to clinically indicated care. Because price-transparency files report payer-negotiated rates rather than out-of-pocket costs, which depend on individual benefit structure, negotiated rates do not directly reflect patient spending but determine the amounts from which many forms of cost sharing are calculated.^59^ These costs may influence care, as most oncologists consider patient out-of-pocket costs for biomarker testing to be an important factor in treatment decisions, and concerns about cost and coverage can influence whether and when testing is pursued.^60^ Such barriers may be consequential given that these tests remain underused, with delays observed among patients of low socioeconomic status.^4,61^ Financial barriers, including variation in patient cost sharing, may contribute to these disparities in access, underscoring the importance of policies that promote adequate insurance coverage for clinically indicated testing. Additional research is needed to determine the extent to which higher prices influence testing access, financial toxicity, or patients' choice of providers or institutions.

Our study suggests heterogeneity in hospital price reporting for precision oncology testing. The Hospital Price Transparency Rule requires hospitals to disclose payer-specific negotiated charges for all items and services for which they have established a standard charge.^30,62^ We found that just over half reported negotiated rates for at least one included biomarker test. Reporting was limited for individual tests, with fewer than one-fifth of hospitals reporting rates for panel-based testing and fewer than one-third reporting rates for non-panel-based testing. However, these findings do not necessarily indicate widespread noncompliance, because not all hospitals perform or bill for oncology biomarker testing. Previous evaluations of compliance with the Hospital Price Transparency Rule similarly demonstrated incomplete reporting of negotiated rates across hospitals and services,^63-66^ although overall price transparency has improved over time, particularly following increases in CMS financial penalties.^63,67^ CMS has finalized additional requirements for 2026, including standardized reporting of allowed amounts and enhanced enforcement, and opportunities exist to require more frequent reporting of biomarker test pricing.^68^ These changes may improve future assessments of pricing variation in oncology care.

In conclusion, this cross-sectional analysis of commercial payer-negotiated rates for precision oncology biomarker tests showed substantial price variation across hospitals and payers. As biomarker testing is increasingly incorporated into cancer care, greater attention to price transparency and efforts to reduce price variation may be necessary to ensure equitable and affordable access to guideline-recommended testing.

## Contribution statement

M.M., J.W.D., and J.S.R. contributed to study concept and design. M.M. abstracted the data, conducted the analyses, and drafted the first version of manuscript. All authors contributed to the analysis and interpretation of the data and the critical revision of the manuscript. JWD provided study supervision.

## Supplementary Material

qxag203_Supplementary_Data

## Data access and responsibility

Dr. Mooghali had full access to all the data in the study and takes responsibility for the integrity of the data and the accuracy of the data analysis.

## Data sharing statement

Relevant data are available on reasonable request from the corresponding author.
