# Supplementary material for: Commercial price variation in precision oncology testing across US hospitals
Source: Health Aff Sch. 2026 Aug 6;4(8):qxag203. doi: 10.1093/haschl/qxag203 (PMC13492076; doi:10.1093/haschl/qxag203)
Supplement: Supplementary file 1 [file qxag203_supplementary_data.zip › qxag203_Supplementary_Data.docx]

**Appendix Table 1– List of Oncology Panel Tests Identified from CMS Clinical Laboratory Fee Schedule**

| **CPT Code** | **Code description as defined by CMS Clinical Laboratory Fee Schedule** |
| --- | --- |
| 0022U | Targeted genomic sequence analysis panel, non-small cell lung neoplasia, dna and rna analysis, 23 genes, interrogation for sequence variants and rearrangements, reported as presence or absence of variants and associated therapy(ies) to consider |
| 0037U | Targeted genomic sequence analysis, solid organ neoplasm, dna analysis of 324 genes, interrogation for sequence variants, gene copy number amplifications, gene rearrangements, microsatellite instability and tumor mutational burden |
| 0045U | Oncology (breast ductal carcinoma in situ), mrna, gene expression profiling by real-time rt-pcr of 12 genes (7 content and 5 housekeeping), utilizing formalin-fixed paraffin-embedded tissue, algorithm reported as recurrence score |
| 0048U | Oncology (solid organ neoplasia), dna, targeted sequencing of protein-coding exons of 468 cancer-associated genes, including interrogation for somatic mutations and microsatellite instability, matched with normal specimens, utilizing formalin-fixed paraffin-embedded tumor tissue, report of clinically significant mutation(s) |
| 0153U | Oncology (breast), mrna, gene expression profiling by next-generation sequencing of 101 genes, utilizing formalin-fixed paraffin-embedded tissue, algorithm reported as a triple negative breast cancer clinical subtype(s) with information on immune cell involvement |
| 0174U | Oncology (solid tumor), mass spectrometric 30 protein targets, formalin-fixed paraffin-embedded tissue, prognostic and predictive algorithm reported as likely, unlikely, or uncertain benefit of 39 chemotherapy and targeted therapeutic oncology agents |
| 0179U | Oncology (non-small cell lung cancer), cell-free dna, targeted sequence analysis of 23 genes (single nucleotide variations, insertions and deletions, fusions without prior knowledge of partner/breakpoint, copy number variations), with report of significant mutation(s) |
| 0244U | Oncology (solid organ), dna, comprehensive genomic profiling, 257 genes, interrogation for single-nucleotide variants, insertions/deletions, copy number alterations, gene rearrangements, tumor-mutational burden and microsatellite instability, utilizing formalin-fixed paraffin-embedded tumor tissue |
| 0249U | Oncology (breast), semiquantitative analysis of 32 phosphoproteins and protein analytes, includes laser capture microdissection, with algorithmic analysis and interpretative report |
| 0250U | Oncology (solid organ neoplasm), targeted genomic sequence dna analysis of 505 genes, interrogation for somatic alterations (snvs [single nucleotide variant], small insertions and deletions, one amplification, and four translocations), microsatellite instability and tumor-mutation burden |
| 0262U | Oncology (solid tumor), gene expression profiling by real-time rt-pcr of 7 gene pathways (er, ar, pi3k, mapk, hh, tgfb, notch), formalin-fixed paraffinembedded (ffpe), algorithm reported as gene pathway activity score |
| 0288U | Oncology (lung), mrna, quantitative pcr analysis of 11 genes (bag1, brca1, cdc6, cdk2ap1, erbb3, fut3, il11, lck, rnd3, sh3bgr, wnt3a) and 3 reference genes (esd, tbp, yap1), formalin-fixed paraffin-embedded (ffpe) tumor tissue, algorithmic interpretation reported as a recurrence risk score |
| 0295U | Oncology (breast ductal carcinoma in situ), protein expression profiling by immunohistochemistry of 7 proteins (cox2, foxa1, her2, ki-67, p16, pr, siah2), with 4 clinicopathologic factors (size, age, margin status, palpability), utilizing formalin-fixed paraffin-embedded (ffpe) tissue, algorithm reported as a recurrence risk score |
| 0332U | Oncology (pan-tumor), genetic profiling of 8 dna-regulatory (epigenetic) markers by quantitative polymerase chain reaction (qpcr), whole blood, reported as a high or low probability of responding to immune checkpoint-inhibitor therapy |
| 0379U | Targeted genomic sequence analysis panel, solid organ neoplasm, dna (523 genes) and rna (55 genes) by next-generation sequencing, interrogation for sequence variants, gene copy number amplifications, gene rearrangements, microsatellite instability, and tumor mutational burden |
| 0388U | Oncology (non-small cell lung cancer), next-generation sequencing with identification of single nucleotide variants, copy number variants, insertions and deletions, and structural variants in 37 cancer-related genes, plasma, with report for alteration detection |
| 0391U | Oncology (solid tumor), dna and rna by next-generation sequencing, utilizing formalin-fixed paraffin-embedded (ffpe) tissue, 437 genes, interpretive report for single nucleotide variants, splicesite variants, insertions/deletions, copy number alterations, gene fusions, tumor mutational burden, and microsatellite instability, with algorithm quantifying immunotherapy response score |
| 0409U | Oncology (solid tumor), dna (80 genes) and rna (36 genes), by next-generation sequencing from plasma, including single nucleotide variants, insertions/deletions, copy number alterations, microsatellite instability, and fusions, report showing identified mutations with clinical actionability |
| 0414U | Oncology (lung), augmentative algorithmic analysis of digitized whole slide imaging for 8 genes (alk, braf, egfr, erbb2, met, ntrk1-3, ret, ros1), and kras g12c and pd-l1, if performed, formalin-fixed paraffin-embedded (ffpe) tissue, reported as positive or negative for each biomarker |
| 0418U | Oncology (breast), augmentative algorithmic analysis of digitized whole slide imaging of 8 histologic and immunohistochemical features, reported as a recurrence score |
| 0444U | Oncology (solid organ neoplasia), targeted genomic sequence analysis panel of 361 genes, interrogation for gene fusions, translocations, or other rearrangements, using dna from formalin-fixed paraffin-embedded (ffpe) tumor tissue, report of clinically significant variant(s) |
| 0473U | Oncology (solid tumor), nextgeneration sequencing (ngs) of dna from formalin-fixed paraffinembedded (ffpe) tissue with comparative sequence analysis from a matched normal specimen (blood or saliva), 648 genes, interrogation for sequence variants, insertion and deletion alterations, copy number variants, rearrangements, microsatellite instability, and tumor-mutation burden |
| 0478U | Oncology (non-small cell lung cancer), dna and rna, digital pcr analysis of 9 genes (egfr, kras, braf, alk, ros1, ret, ntrk 1/2/3, erbb2, and met) in formalin-fixed paraffin-embedded (ffpe) tissue, interrogation for single-nucleotide variants, insertions/deletions, gene rearrangements, and reported as actionable detected variants for therapy selection |
| 0487U | Oncology (solid tumor), cell-free circulating dna, targeted genomic sequence analysis panel of 84 genes, interrogation for sequence variants, aneuploidycorrected gene copy number amplifications and losses, gene rearrangements, and microsatellite instability |
| 0499U | Oncology (colorectal and lung), dna from formalin-fixed paraffinembedded (ffpe) tissue, nextgeneration sequencing of 8 genes (nras, egfr, ctnnb1, pik3ca, apc, braf, kras, and tp53), mutation detection |
| 0523U | Oncology (solid tumor), dna, qualitative, next-generation sequencing (ngs) of single-nucleotide variants (snv) and insertion/deletions in 22 genes utilizing formalin-fixed paraffin-embedded tissue, reported as presence or absence of mutation(s), location of mutation(s), nucleotide change, and amino acid change |
| 0530U | Oncology (pan-solid tumor), ctdna, utilizing plasma, next-generation sequencing (ngs) of 77 genes, 8 fusions, microsatellite instability, and tumor mutation burden, interpretative report for single-nucleotide variants, copy-number alterations, with therapy association |
| 0538U | Oncology (solid tumor), nextgeneration targeted sequencing analysis, formalin-fixed paraffinembedded (ffpe) tumor tissue, dna analysis of 600 genes, interrogation for single-nucleotide variants, insertions/deletions, gene rearrangements, and copy number alterations, microsatellite instability, tumor mutation burden, reported as actionable variant |
| 0539U | Oncology (solid tumor), cellfree circulating tumor dna (ctdna), 152 genes, nextgeneration sequencing, interrogation for singlenucleotide variants, insertions/deletions, gene rearrangements, copy number alterations, and microsatellite instability, using whole-blood samples, mutations with clinical actionability reported as actionable variant |
| 0543U | Oncology (solid tumor), nextgeneration sequencing of dna from formalin-fixed paraffin-embedded (ffpe) tissue of 517 genes, interrogation for single-nucleotide variants, multinucleotide variants, insertions and deletions from dna, fusions in 24 genes and splice variants in 1 gene from rna, and tumor mutation burden |
| 0562U | Oncology (solid tumor), targeted genomic sequence analysis, 33 genes, detection of single-nucleotide variants (snvs), insertions and deletions, copy-number amplifications, and translocations in human genomic circulating cell-free dna, plasma, reported as presence of actionable variants |
| 0571U | Oncology (solid tumor), dna (80 genes) and rna (10 genes), by next-generation sequencing, plasma, including single-nucleotide variants, insertions/deletions, copy-number alterations, microsatellite instability, and fusions, reported as clinically actionable variants |
| 0585U | Targeted genomic sequence analysis panel, solid organ neoplasm, circulating cell-free dna (cfdna) analysis from plasma of 521 genes, interrogation for sequence variants, gene copy number amplifications, gene rearrangements, and microsatellite instability, report shows identified mutations, including variants with clinical actionability |
| 0597U | Oncology (breast), rna expression profiling of 329 genes by targeted nextgeneration sequencing and 20 proteins by multiplex immunofluorescence, formalin-fixed paraffinembedded (ffpe) tissue, algorithmic analyses to determine tumor-recurrence risk score |
| 81523 | Oncology (breast), mrna, next-generation sequencing gene expression profiling of 70 content genes and 31 housekeeping genes, utilizing formalin-fixed paraffin-embedded tissue, algorithm reported as index related to risk to distant metastasis |
| 81525 | Oncology (colon), mrna, gene expression profiling by real-time rt-pcr of 12 genes (7 content and 5 housekeeping), utilizing formalin-fixed paraffin-embedded tissue, algorithm reported as a recurrence score |
| 81538 | Oncology (lung), mass spectrometric 8-protein signature, including amyloid a, utilizing serum, prognostic and predictive algorithm reported as good versus poor overall survival |

Source: Authors’ analysis of data from the CMS Clinical Laboratory Fee Schedule

Note: CPT, Current Procedural Terminology; CMS, Centers for Medicare & Medicaid Services.

**Appendix Table 2 – NCCN Biomarkers Compendium-Recommended Biomarker Tests Without Unique CPT Codes (Excluded from Sample)**

| **Biomarker Tests** | **Indication** |
| --- | --- |
| ALK fusion testing | Non-Small Cell Lung Cancer |
| PD-L1 expression | Breast Cancer, Non-Small Cell Lung Cancer |
| ERBB2/HER2 mutation/amplification | Non-Small Cell Lung Cancer, Breast Cancer, Colon Cancer |
| FGFR alterations | Non-Small Cell Lung Cancer, Breast Cancer |
| MET mutation/amplification | Non-Small Cell Lung Cancer |
| NRG1 gene rearrangement | Non-Small Cell Lung Cancer |
| NUTM1 fusion | Non-Small Cell Lung Cancer |
| Pankeratin AE1/AE3 expression | Non-Small Cell Lung Cancer |
| RET gene rearrangement | Non-Small Cell Lung Cancer, Breast Cancer, Colon Cancer |
| ROS1 gene rearrangement | Non-Small Cell Lung Cancer |
| Expression of markers to distinguish mesothelioma and metastatic carcinoma: CDX2, ESR1, GATA3, NAPSA, NKX2-1, NKX3-1, PAX2, PAX8, PGR, PIP, SCGB2A2 | Non-Small Cell Lung Cancer |
| Immunophenotype (NAPSA, NKX2-1, TP63) | Non-Small Cell Lung Cancer |
| Immunophenotype (CHGA, INSM1, NCAM1, SYP) | Non-Small Cell Lung Cancer |
| Immunophenotype (CALB2, KRT5, KRT6A, KRT6B, PDPN, WT1) | Non-Small Cell Lung Cancer |
| Immunophenotype (CEACAM5, CLDN4, EPCAM, FUT4, NAPSA, NKX2-1) | Non-Small Cell Lung Cancer |
| Immunophenotype (PAX8, TG) | Non-Small Cell Lung Cancer |
| AKT1, PIK3CA, PTEN mutation testing | Breast Cancer |
| POLE/POLD1 mutation | Colon Cancer |
| TMB (tumor mutation burden) | Breast Cancer |

Source: Authors’ analysis of data from the NCCN Biomarkers Compendium.

Notes: CPT, Current Procedural Terminology; NCCN, National Comprehensive Cancer Network.

**Appendix Table 3 – List of Unique CPT codes associated with the NCCN Biomarkers Compendium-Recommended Biomarker Tests**

| **CPT Code** | **Code description as defined by CMS Clinical Laboratory Fee Schedule** | **Indication** | **NCCN Category of Evidence** |
| --- | --- | --- | --- |
| 81210 | BRAF (b-raf proto-oncogene, serine/threonine kinase) (eg, colon cancer, melanoma), gene analysis, v600 variant(s) | Non-Small Cell Lung Cancer, Colon Cancer | Category 2A |
| 81162 | BRCA 1 (brca1, dna repair associated), brca2 (brca2, dna repair associated) (eg, hereditary breast and ovarian cancer) gene analysis; full sequence analysis and full duplication/deletion analysis (ie, detection of large gene rearrangements) | Breast Cancer | Category 2A |
| 81163 | BRCA 1 (brca1, dna repair associated), brca2 (brca2, dna repair associated) (eg, hereditary breast and ovarian cancer) gene analysis; full sequence analysis | Breast Cancer | Category 2A |
| 81164 | BRCA 1 (brca1, dna repair associated), brca2 (brca2, dna repair associated) (eg, hereditary breast and ovarian cancer) gene analysis; full duplication/deletion analysis (ie, detection of large gene rearrangements) | Breast Cancer | Category 2A |
| 81165 | BRCA 1 (brca1, dna repair associated) (eg, hereditary breast and ovarian cancer) gene analysis; full sequence analysis | Breast Cancer | Category 2A |
| 81166 | BRCA 1 (brca1, dna repair associated) (eg, hereditary breast and ovarian cancer) gene analysis; full duplication/deletion analysis (ie, detection of large gene rearrangements) | Breast Cancer | Category 2A |
| 81167 | BRCA 2 (brca2, dna repair associated) (eg, hereditary breast and ovarian cancer) gene analysis; full duplication/deletion analysis (ie, detection of large gene rearrangements) | Breast Cancer | Category 2A |
| 81212 | BRCA 1 (brca1, dna repair associated), brca2 (brca2, dna repair associated) (eg, hereditary breast and ovarian cancer) gene analysis; 185delag, 5385insc, 6174delt variants | Breast Cancer | Category 2A |
| 81215 | BRCA 1 (brca1, dna repair associated) (eg, hereditary breast and ovarian cancer) gene analysis; known familial variant | Breast Cancer | Category 2A |
| 81216 | BRCA 2 (brca2, dna repair associated) (eg, hereditary breast and ovarian cancer) gene analysis; full sequence analysis | Breast Cancer | Category 2A |
| 81217 | BRCA2 (brca2, dna repair associated) (eg, hereditary breast and ovarian cancer) gene analysis; known familial variant | Breast Cancer | Category 2A |
| 82378 | Carcinoembryonic antigen (CEA) | Breast Cancer, Colon Cancer | Category 2A |
| 81232 | DPYD (dihydropyrimidine dehydrogenase) (eg, 5-fluorouracil/5-fu and capecitabine drug metabolism), gene analysis, common variant(s) (eg, *2a, *4, *5, *6) | Colon Cancer | Category 2A |
| 81235 | EGFR (epidermal growth factor receptor) (e.g., non-small cell lung cancer) gene analysis, common variants (e.g., exon 19 LREA deletion, L858R, T790M, G719A, G719S, L861Q) | Non-Small Cell Lung Cancer | Category 1 for certain indications, and Category 2A for others |
| 84233 | Receptor assay; estrogen | Breast Cancer | Category 2A |
| 81522 | Oncology (breast), mRNA, gene expression profiling by RT-PCR of 12 genes (8 content and 4 housekeeping), utilizing formalin-fixed paraffin-embedded tissue, algorithm reported as recurrence risk score | Breast Cancer | Category 2A |
| 81521 | Oncology (breast), mrna, microarray gene expression profiling of 70 content genes and 465 housekeeping genes, utilizing fresh frozen or formalin-fixed paraffin-embedded tissue, algorithm reported as index related to risk of distant metastasis | Breast Cancer | Category 1 |
| 81520 | Oncology (breast), mRNA gene expression profiling by hybrid capture of 58 genes (50 content and 8 housekeeping), utilizing formalin-fixed paraffin-embedded tissue, algorithm reported as a recurrence risk score | Breast Cancer | Category 2A |
| 81518 | Oncology (breast), mRNA, gene expression profiling by real-time RT-PCR of 11 genes (7 content and 4 housekeeping), utilizing formalin-fixed paraffin-embedded tissue, algorithms reported as percentage risk for metastatic recurrence and likelihood of benefit from extended endocrine therapy | Breast Cancer | Category 2A |
| 81519 | Oncology (breast), mRNA, gene expression profiling by real-time RT-PCR of 21 genes, utilizing formalin-fixed paraffin-embedded tissue, algorithm reported as recurrence score | Breast Cancer | Category 1 |
| 81275 | KRAS (kirsten rat sarcoma viral oncogene homolog) (eg, carcinoma) gene analysis; variants in exon 2 (eg, codons 12 and 13) | Colon Cancer | Category 2A |
| 81276 | KRAS (kirsten rat sarcoma viral oncogene homolog) (eg, carcinoma) gene analysis; additional variant(s) (eg, codon 61, codon 146) | Colon Cancer | Category 2A |
| 0111U | Oncology (colon cancer), targeted KRAS (codons 12, 13, and 61) and NRAS (codons 12, 13, and 61) gene analysis utilizing formalin-fixed paraffin-embedded tissue | Colon Cancer | Category 2A |
| 0471U | Oncology (colorectal cancer), 35 variants in KRAS and NRAS genes | Colon Cancer | Category 2A |
| 81301 | Microsatellite instability analysis (e.g., hereditary non-polyposis colorectal cancer, Lynch syndrome) of markers for mismatch repair deficiency (e.g., BAT25, BAT26), includes comparison of neoplastic and normal tissue, if performed | Breast Cancer, Colon Cancer | Category 2A |
| 0158U | MLH1 (mutl homolog 1) (eg, hereditary non-polyposis colorectal cancer, lynch syndrome) mrna sequence analysis (list separately in addition to code for primary procedure) | Breast Cancer, Colon Cancer | Category 2A |
| 0159U | MSH2 (muts homolog 2) (eg, hereditary colon cancer, lynch syndrome) mrna sequence analysis (list separately in addition to code for primary procedure) | Breast Cancer, Colon Cancer | Category 2A |
| 0160U | MSH6 (muts homolog 6) (eg, hereditary colon cancer, lynch syndrome) mrna sequence analysis (list separately in addition to code for primary procedure) | Breast Cancer, Colon Cancer | Category 2A |
| 0161U | PMS2 (pms1 homolog 2, mismatch repair system component) (eg, hereditary nonpolyposis colorectal cancer, lynch syndrome) mrna sequence analysis (list separately in addition to code for primary procedure) | Breast Cancer, Colon Cancer | Category 2A |
| 86300 | Immunoassay for tumor antigen, quantitative; CA 15-3 and CA 27.29 | Breast Cancer | Category 2A |
| 81191 | NTRK1 (neurotrophic receptor tyrosine kinase 1) (eg, solid tumors) translocation analysis | NSCLC, Breast Cancer | Category 2A |
| 81192 | NTRK2 (neurotrophic receptor tyrosine kinase 2) (eg, solid tumors) translocation analysis | NSCLC, Breast Cancer | Category 2A |
| 81193 | NTRK3 (neurotrophic receptor tyrosine kinase 3) (eg, solid tumors) translocation analysis | NSCLC, Breast Cancer | Category 2A |
| 81194 | NTRK (neurotrophic receptor tyrosine kinase 1, 2, and 3) (eg, solid tumors) translocation analysis | NSCLC, Breast Cancer | Category 2A |
| 81307 | PALB2 (partner and localizer of BRCA2) (e.g., breast and pancreatic cancer) gene analysis, full gene sequence | Breast Cancer | Category 2A |
| 81308 | PALB2 (partner and localizer of BRCA2) (e.g., breast and pancreatic cancer) gene analysis, known familial variant | Breast Cancer | Category 2A |
| 0137U | PALB2 (partner and localizer of BRCA2) (eg, breast and pancreatic cancer) mrna sequence analysis (list separately in addition to code for primary procedure) | Breast Cancer | Category 2A |
| 84234 | Assay of progesterone | Breast Cancer | Category 2A |
| 81309 | PIK3CA (phosphatidylinositol-4,5-bisphosphate 3-kinase, catalytic subunit alpha) (e.g., colorectal and breast cancer) gene analysis, targeted sequence analysis (e.g., exons 7, 9, 20) | Breast Cancer, Colon Cancer | Category 2A |
| 0155U | Oncology (breast cancer), DNA, PIK3CA (phosphatidylinositol-4,5-bisphosphate 3-kinase, catalytic subunit alpha) (e.g., breast cancer) gene analysis (i.e., P.C420R, P.E542K, P.E545A, P.E545D [G.1635G>T only], P.E545G, P.E545K, P.Q546E, P.Q546R, P.H1047L, P.H1047R, P.H1047Y), utilizing formalin-fixed paraffin-embedded breast tumor tissue, reported as PIK3CA gene mutation status | Breast Cancer, Colon Cancer | Category 2A |
| 0177U | Oncology (breast cancer), dna, pik3ca (phosphatidylinositol-4,5-bisphosphate 3-kinase catalytic subunit alpha) gene analysis of 11 gene variants utilizing plasma, reported as pik3ca gene mutation status | Breast Cancer, Colon Cancer | Category 2A |

Source: Authors’ analysis of data from the NCCN Biomarkers Compendium.

Notes: CPT, Current Procedural Terminology; NCCN, National Comprehensive Cancer Network.

**Appendix Figure 1– Sample Construction for Biomarkers Tests Used in Precision Oncology Testing**

Notes: CPT, Current Procedural Terminology; CMS, Centers for Medicare & Medicaid Services; NCCN, National Comprehensive Cancer Network

**Appendix Table 4 – Price Variability of Biomarker Tests Used in Precision Oncology Based on Rurality**

| **Biomarker Test (CPT Code)** | **Rurality** | **Mean Difference, USD (95% CI)** | **P value** | **FDR-adjusted Overall P value** |
| --- | --- | --- | --- | --- |
| ***Panel-based Biomarker Tests*** | | | | |
| Breast Cancer Index (81518) | Rural | Reference | Not applicable | 0.96 |
|  | Urban | $-294 (-1,163 to 574) | 0.507 |  |
| EndoPredict, 12 gene (81522) | Rural | Reference | Not applicable | 0.96 |
|  | Urban | $-78 (-1,009 to 854) | 0.87 |  |
| FoundationOne, 324-gene (0037U) | Rural | Reference | Not applicable | 0.75 |
|  | Urban | $677 (-23 to 1,376) | 0.058 |  |
| MammaPrint, 70-gene (81521) | Rural | Reference | Not applicable | 0.96 |
|  | Urban | $-180 (-1,059 to 700) | 0.689 |  |
| MSK-IMPACT, 468-gene (0048U) | Rural | Reference | Not applicable | 0.96 |
|  | Urban | $-122 (-827 to 582) | 0.733 |  |
| Oncotype DX Breast Recurrence Score, 21-gene (81519) | Rural | Reference | Not applicable | 0.96 |
|  | Urban | $-62 (-832 to 709) | 0.875 |  |
| Oncotype DX Colon Cancer Assay, 12-gene (81525) | Rural | Reference | Not applicable | 0.96 |
|  | Urban | $-333 (-1,036 to 371) | 0.354 |  |
| Oncotype DX DCIS Score, 12-gene (0045U) | Rural | Reference | Not applicable | 0.96 |
|  | Urban | $-29 (-964 to 905) | 0.951 |  |
| Oncotype MAP PanCancer Tissue Test, 257-gene (0244U) | Rural | Reference | Not applicable | 0.96 |
|  | Urban | $-22 (-878 to 834) | 0.96 |  |
| Oncomine™ Dx Target Test, 23-gene (0022U) | Rural | Reference | Not applicable | 0.96 |
|  | Urban | $-308 (-777 to 160) | 0.197 |  |
| PGDx elio™ Tissue Complete, 505-gene (0250U) | Rural | Reference | Not applicable | 0.96 |
|  | Urban | $106 (-606 to 817) | 0.771 |  |
| Prosigna, 50-gene (81520) | Rural | Reference | Not applicable | 0.96 |
|  | Urban | $-25 (-627 to 577) | 0.936 |  |
| VeriStrat, 8-protein (81538) | Rural | Reference | Not applicable | 0.96 |
|  | Urban | $-303 (-981 to 375) | 0.381 |  |
| ***Non-panel-based Biomarker Tests*** | | | | |
| *BRAF* mutation (81210) | Rural | Reference | Not applicable | 0.77 |
|  | Urban | $7 (-19 to 33) | 0.592 |  |
| *BRCA1/2* mutation (81162) | Rural | Reference | Not applicable | 0.71 |
|  | Urban | $104 (-178 to 385) | 0.471 |  |
| *CA15-3* or *CA27.29* expression (86300) | Rural | Reference | Not applicable | 0.77 |
|  | Urban | $1 (-3 to 4) | 0.774 |  |
| *CEA* expression (82378) | Rural | Reference | Not applicable | 0.47 |
|  | Urban | $-2 (-6 to 1) | 0.249 |  |
| *DPYD* variant testing (81232) | Rural | Reference | Not applicable | 0.17 |
|  | Urban | $26 (-2 to 55) | 0.068 |  |
| *EGFR* mutation (81235) | Rural | Reference | Not applicable | 0.47 |
|  | Urban | $27 (-19 to 72) | 0.248 |  |
| Estrogen receptor expression (84233) | Rural | Reference | Not applicable | 0.17 |
|  | Urban | $16 (0 to 32) | 0.046 |  |
| *KRAS* mutation (81275) | Rural | Reference | Not applicable | 0.17 |
|  | Urban | $26 (-2 to 53) | 0.065 |  |
| *KRAS/NRAS* mutation (0111u) | Rural | Reference | Not applicable | 0.77 |
|  | Urban | $-27 (-189 to 136) | 0.749 |  |
| Mismatch repair testing (0160u) | Rural | Reference | Not applicable | 0.17 |
|  | Urban | $67 (-4 to 138) | 0.065 |  |
| Microsatellite instability testing (81301) | Rural | Reference | Not applicable | 0.04 |
|  | Urban | $75 (27 to 123) | 0.002 |  |
| *NTRK* gene fusion testing (81194) | Rural | Reference | Not applicable | 0.77 |
|  | Urban | $22 (-84 to 129) | 0.682 |  |
| *PALB2* mutation (81307) | Rural | Reference | Not applicable | 0.77 |
|  | Urban | $36 (-103 to 174) | 0.614 |  |
| *PIK3CA* mutation (81309) | Rural | Reference | Not applicable | 0.70 |
|  | Urban | $24 (-34 to 82) | 0.42 |  |
| Progesterone receptor expression (84234) | Rural | Reference | Not applicable | 0.04 |
|  | Urban | $18 (6 to 31) | 0.005 |  |

Source: Authors’ analysis of data from the Turquoise Health hospital pricing database.

Notes: CPT, Current Procedural Terminology; FDR, False Discovery Rate; IQR, interquartile range; USD, US Dollar.
